# Towards a process-oriented understanding of HR analytics: implementation and application

**DOI:** 10.1007/s11846-022-00574-0

**Published:** 2022-08-18

**Authors:** Felix Wirges, Anne-Katrin Neyer

**Affiliations:** Chair of Human Resources Management & Business Governance, Martin-Luther-University Halle-Wittenberg, Halle, Germany

**Keywords:** HR analytics, People analytics, HR-data, HR-metrics, M1, M12, M15

## Abstract

Firms have recognized the opportunities presented by HR analytics; however, it is challenging for HR to convert their available data (sources) into meaningful strategical value. Moreover, research on the implementation and application of HR analytics is still in its infancy. Drawing on the socio-technical system perspective, we examine the implementation and application of HR analytics in firms. Based on a qualitative study with 17 HR analytics experts, we find that a shift to a more process-oriented perspective on HR analytics is needed. More precisely, besides the requirements for the analysis of data, the actual roles in the process of implementing and applying HR analytics need to be defined. In particular, this implies the interaction between the specialist department, the HR business partner and the HR analytics function. From a managerial perspective, we propose a process model for the future implementation and application of HR analytics.

## Introduction

Data collection and analysis is an essential part of business process decision-making for a variety of organizations (Côrte-Real et al. 2017; George et al. 2014). Data-based decision-making takes place in almost every area of an organization; marketing, sales, production and finance are examples of application areas where it is common to make decisions based on analysis and reporting. This is not particularly surprising, as analytics-based decision-making can automate processes and make them more efficient in almost every business area where data can be gathered (Acito and Khatri 2014; Earley 2015; Ghasemaghaei 2018).

However, compared to the extensive use of data in other application areas of the company, its use is rather rare in HR (Tursunbayeva et al. 2018). To help to deal with this endeavor, in recent years, the term "HR analytics" has become popular and has increasingly been used in science and practice, often promising nothing less than the revolving of the HR management (Marler and Boudreau 2017; van den Heuvel and Bondarouk 2017; Huselid 2018; McIver et al. 2018; Tursunbayeva et al. 2018; Greasley and Thomas 2020; McCartney and Fu 2022). Falletta and Combs (2021) note that while the amount of data and technology has increased significantly, the use of data to explain organizational issues is not in itself a novel tool. A variety of approaches exist, focusing on a more evidence-based approach to HR management (Lawler and Boudreau 2015). This emphasizes that the design of the HR function is per se predestinated for the use of data-based decisions:

First, HR is a business area that, in principle, could generate a large amount of data. Through the ongoing digitization of work processes, the use of mobile devices, the wearing of wearables or the use of company apps, employees generate different types of data. These include e.g. information about locations, communication, personal well-being and many other factors, which are of relevance for HR (Cascio and Montealegre 2016).

Second, due to the growing use of information and communication technologies in HR, the term eHRM has become increasingly popular (van den Heuvel and Bondarouk 2017). More recently, the use of HR analytics has taken the topic to a new level: The previous use of technology in HR mostly concentrated on operative support or descriptive reporting of key information, such as sick days of the workforce or employee turnover. Through the use of HR analytics, connections and conclusions can now be drawn (a) in each functional area of HR, but also (b) with data from other application areas. Predictive analyses can then be made from these results. However, if we look at the practical application of HR analytics, especially with a focus on the use of predictive analytics, we see a sobering picture. Falletta (2014) shows in a sample of 220 firms that only 15% place a strategic focus on HR analytics and that, as a rule, they do not carry out any predictive analyses, but only focus on reporting. A survey by Lawler and Boudreau (2015) published two years later provides similar results. Levenson and Fink (2017) noted that the term HR analytics has become a catch-all term to describe any handling of data and metrics in HR: “It has come to include anything numerical about talent and HR work. Examples include simple data reports, analyzing data integrated from multiple systems (e.g. performance and compensation), dashboards, making data available “on demand,” and true talent or “predictive” analytics” (Levenson and Fink 2017:146). More recent definitions of HR analytics emphasize shifting the focus towards a process perspective (Mclver et al. 2018): HR analytics is not only understood as a tool where statistical methods are applied and the focus is on key figures, but as a systematic approach (Falletta and Combs 2021). Most recently, Falletta and Combs (2021) define HR analytics as follows: “HR analytics is a proactive and systematic process for ethically gathering, analyzing, communicating and using evidence-based HR research and analytical insights to help organizations achieve their strategic objectives” (Falletta and Combs 2021: 3). The two authors highlight that in the application of HR analytics so far, there is too little recognition “of the role of broader HR research and experimentation as part of an overarching HR analytics agenda (i.e. internal HR research or partnership research in the context of social, behavioral and organizational sciences)” (Falletta and Combs 2021: 54). This goes in line with the evidence-based review by Marler and Boudreau (2017), which uses an integrative synthesis of published peer-reviewed literature. Their findings emphasize that HR could soon be technically left behind and thus, hint at an issue that has already been discussed for some years: HR must create technological change in order to continue to play an equal role in the company in the future (Shrivastava and Shaw 2003; Snell et al. 2002; Ulrich 1997).

Third, in the wake of the COVID-19 pandemic, it became clear that metrics-based information can be of tremendous importance. Companies were confronted overnight with unprecedented challenges in managing employees in the workplace. The HR function in particular had to ensure within a very short time that the new requirements for remote work, digital collaboration and leadership in teams, and (mental) health issues were met (Kniffin et al. 2020). Along the way, previous tasks such as recruiting or HR development had to be transformed into digital solutions. Companies, therefore, raised information about remote work, employee engagement, and well-being to gain a clear picture of the respective needs of employees (Belizón and Kieran 2021). The situation created by the pandemic highlights the importance of HR analytics. It essentially offers the possibility of many new types of data sources that can specifically promote the quality of HR analytics (Bryce et al. 2022).

Having said this, the pressing issue thus is: why do only a few organizations rely on the (advanced) use of HR analytics, although the circumstances (data generation, software applications, etc.) and the reason (strengthening the strategic role of HR) (Bassi et al. 2012) seem to be predestined for an application? Building on this question, we conducted an initial descriptive survey with HR employees and managers to determine the status quo of the implementation of data-based analytics (Wirges et al. 2020) (see Table 1).

**Table 1:** Framework data of the HR study 2020

Participants	117 employees and managers HR, commercial management and the executive board
Sector groups	Three largest sector groups 20% information and communication technology 17% industrial and industrial services 13% health care and pharmaceutical industry
Firm size	26% up to 200 16% up to 500 14% up to 1000 20% up to 5000 6% up to 10,000 and 17% over 10,000 employees
Period	October 2019 until January 2020

The results of the study showed that working with data plays an important role in HR management. 66% of the interviewees said that data evaluation in HR is included in strategic decision-making. However, it should be noted that these decisions are predominantly based on classic HR controlling (Excel, KPI etc.) or descriptive analyses (the analysis of data related to the past). For example, 96% of the interviewees stated that they use the data obtained for HR controlling. In this study, HR controlling was broadly defined as the simple collection and reporting of individually defined key performance indicators. The use for descriptive analyses is already significantly lower at 32%. Only 5% carry out predictive analyses with the help of the data.

The results of this study reflect a sobering picture regarding the application of HR analytics. This underlines the current state in the literature that the knowledge about the implementation and application of HR analytics is fraught with many challenges and difficulties. For a better understanding of these challenges and to present them in a holistic picture, the aim of this paper is to dive deeper into the implementation and application of HR analytics. To do so, we conducted a qualitative study with HR analytics experts. By applying a socio-technical approach as a theoretical lens, we aim to answer the following research questions: How is the implementation and application of HR analytics shaping up in firms? What challenges and barriers do firms face on their journey towards HR analytics?

## Theoretical background

To answer our research questions, we apply a socio-technical approach. Socio-technical system theory assumes that new systems can only be successful if both the technical and the social system are considered, analyzed and brought into harmony with each other on an equal footing (Cherns 1976). The social system describes the people in an organization and focuses on their needs, relationships and qualifications within the organization. The technical system, on the other hand, often describes novel technological artifacts used to accomplish tasks (Jaffee 2001; Mumford 2003). The socio-technical paradigm is a holistic view that examines the relationships between the social and technical levels of any system (Trist and Bamforth 1951; Coakes 2002). Socio-technical design emphasizes the need for an optimal match between the technical and social aspects in terms of the relationship between jobs and people's needs and expectations (Biazzo 2002). As discussed earlier, understanding HR analytics from a systematic process perspective has gained importance (e.g. Falletta and Combs 2021). In the literature to date, there are initial approaches to linking the socio-technical approach in the context of HR analytics (Belizón and Kieran 2021). Nevertheless, this can be classified as rather novel. Thereby, the goal of HR analytics is to enable organizations to achieve their strategic objectives. Since HR analytics comprises more than the introduction of software for personal data analysis, it requires a more holistic approach rather than a traditional IT project approach. Often, projects of this kind fail not because of the technology, but because of a lack of consideration of the mutual interactions of the social and technical system. Maucher et al. (2002) show that soft factors such as communication, cooperation and the inclusion of informal structures can contribute significantly to the successful implementation and application of IT projects.

**Fig. 1:**
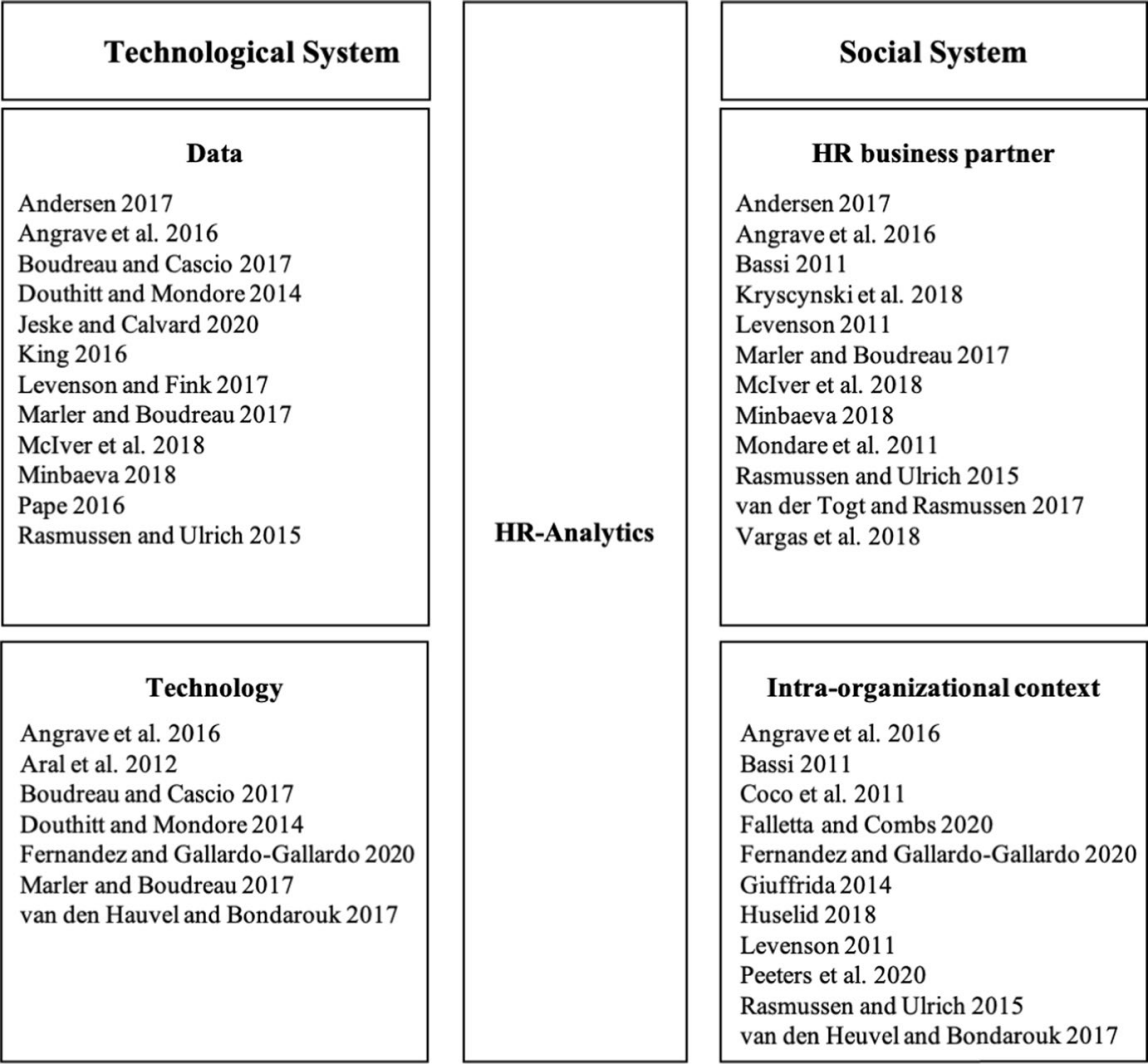
Core areas influencing HR analytics

Following the socio-technical system theory proposal we analyze the extent to which the technological side, i.e. the artifact needed for the data analysis and the social side, i.e. the organizational structures of a company with a large number of different stakeholders, need to be reflected by HR analytics. We conducted a literature review and classified the challenges we found for the implementation and application of HR analytics within the sociotechnical perspective into the two categories of social and technological. This involved searching for peer-reviewed journal articles that address challenges in the implementation and application of HR analytics. For this purpose, the following search terms were used to identify relevant articles: "HR analytics"; "People analytics"; "Human resource analytics"; "Workforce analytics"; "Data-driven HR" in combination with "challenges"; "difficulties"; and "barriers". The focus in the selection of the respective journal articles was on the thematization of concrete examples of implementation and application difficulties. In doing so, we were able to identify four core areas (see Fig. 1) that influence the implementation and application of HR analytics from a systematic process perspective.

The first of the areas of the technological system we examine involves the data, which represent the elementary cornerstones for carrying out the analyses (Douthitt and Mondore 2014; Pape 2016). One is the quantitative aspect of analyzing whether or not the necessary HR databases with the associated data sources exist for the use of HR analytics. On the other hand, it must be seen whether the existing data meet the qualitative requirements in order to be able to carry out valid analyses (Jeske and Calvard 2020; King 2016; Minbaeva 2018; Pape 2016). Previous research shows the need for the integration of additional data from different areas of the company into the analysis (Marler and Boudreau 2017; McIver et al. 2018; Rasmussen and Ulrich 2015). To do so, interface compatibility plays an important role (Andersen 2017; Boudreau and Cascio 2017; Douthitt and Mondore 2014; Levenson and Fink 2017). Angrave et al. (2016) emphasize this by warning against data silos formed within the individual departments leading to a lack of data exchange. Indeed, the lack of available data has been identified as one of the main obstacles to the successful implementation of analytics, especially in small and medium-sized enterprises (Pape 2016).

The second area at the technological level comprises the technology itself i.e. which software and hardware solutions are available to users (Angrave et. al 2016; Aral et al. 2012; Boudreau and Cascio 2017; Douthitt and Mondore 2014). A lot of firms still stick to simple spreadsheet programs such as Excel for data analysis (van den Hauvel and Bondarouk 2017), even though in recent years the number of other tool providers, offering a wider range of functions for data analysis, has increased. However, so far, the available tools for predictive and prescriptive HR analytics are developed by and aimed at people with analytical skills, not HR business partners (Fernandez and Gallardo-Gallardo 2021). This is also shown by the results of our descriptive study, in which we found that the available software solutions are perceived to be too complex. More precisely, software solutions for the application of HR analytics are not tailored to the competencies of the users and, thus, will need to be much more user-friendly (Marler and Boudreau 2017). The application of the technology, i.e., software solutions, therefore depends strongly on the respective competencies of the HR business partners or the users of HR analytics in a company.

This assumption simultaneously emphasizes the interconnection of the technological with the social system. A strong social system lays the foundations for the implementation and application of HR analytics. Within the social system, we first focus on the HR business partner as a user of HR analytics (Bassi 2011; Mondare et al. 2011; Rasmussen and Ulrich 2015; Angrave et al. 2016). It is generally agreed that one of the main reasons for the low use of HR analytics is the lack of analytical skills (Angrave et al. 2016; Marler and Boudreau 2017). However, these analytical skills are an elementary prerequisite for performing HR analytics (Andersen 2017; Douthitt and Carson 2011; Huselid 2018; Kryscynski et al. 2018, Marler and Boudreau 2017, Minbaeva 2018, van der Togt and Rasmussen 2017). Prior studies have analyzed that individuals working in HR are not primarily interested in operating with key figures, statistical methods or data analysis (Rasmussen and Ulrich 2015). Fernandez and Gallardo-Gallardo (2021) emphasize that the analytical skills needed to apply HR analytics will increase in the future. Thus, a wider range of basic statistical methods in the individual maturity levels of data analysis (reporting, descriptive, predictive, prescriptive) and analytical competencies in data collection and data management are considered important to HR analytics (Levenson 2011). Because of the advancing increase in artificial intelligence and its methods such as machine learning, we assume that HR business partners will be required to continuously learn and extend their knowledge (McIver et al. 2018). The personnel development measures required for this in turn have a positive effect on the attitude toward HR analytics and increase the individual's self-efficacy (Vargas et al. 2018).

The intra-organizational context is also relevant in explaining the influence of the social system on the implementation and application of HR analytics. First, the focus is on the different stakeholders involved in the process (Coco et al. 2011; Giuffrida 2014; Levenson 2011; Rasmussen and Ulrich 2015). These can be divided into HR business partners, management, employees and analysis teams (Huselid 2018; Peeters Paauwe and van de Voorde 2020). In previous research, there are few findings about which stakeholders are involved in the process of HR analytics (Coco et al. 2011). However, we argue that our analysis shows they do not look at the respective roles and relationships in the implementation and application of HR analytics in an organization. Second, there are two perspectives that explain how HR analytics should be embedded within the organization, i.e. outsourcing and integration (van den Heuvel and Bondarouk 2017). The two different perspectives can be characterized as follows: In outsourcing, the analyses are carried out by experts in the analysis area and not by the actual HR business partners. The HR analytics function operates independently alongside the traditional HR function (Fernandez and Gallardo-Gallardo 2021; Rasmussen and Ulrich 2015). In the case of integration, an attempt is made to strengthen the competencies of the HR business partner and to perform the analyses within the HR function with the help of HR analytics. Outsourcing HR analytics from the HR function is supposed to align HR analytics more strategically (Rasmussen and Ulrich 2015). In contrast, its integration into the HR function (Angrave et al. 2016; Bassi 2011; Falletta and Combs 2021), will enable HR to strengthen its own strategic role. Additionally, it is argued that the expertise of HR business partners in the respective functional areas is needed. If they are not directly integrated into the process, the usefulness of the analysis of HR-specific issues can only be assessed to a limited extent (Andersen 2017).

Having presented our framework, we follow Greasley and Thomas (2020) call for further empirical analysis of analytics projects to conduct research in HR analytics with a focus “on the process of development rather than its outcomes” (Greasley and Thomas 2020: 506).

## Methodological approach

In order to gain deeper insights into the implementation and application of HR analytics, we conducted a qualitative study with 17 HR analytics experts from the DACH region. Our study aims to understand the process of implementing and using HR analytics in more detail Therefore, we use a qualitative research approach, which is particularly suitable for investigating topics that have been little empirically researched so far and require a deeper insight into situational conditions. Moreover, qualitative research also lends itself specifically to the representation of organizational processes, as one can derive important information about social interactions and causal relationships from the depth and variety of data obtained (Graebner et al. 2012).

Hyde (2000) notes that the information content in qualitative research is based on the depth of the interviews and even the knowledge of one person, if the rules of qualitative social research are followed, can provide insightful knowledge about complex issues (Hyde 2000). Thus, the identification of the experts was the first crucial step in our study. A targeted search was conducted via job-related social networks for job titles that included the competence profile HR analytics, people analytics, workforce analytics or HR executives who mentioned working with data in the HR management in their competence profile. To make sure that only HR analytics experts take part in the qualitative study, the selection of participants study was based on the following criteria:

1. The interviewees explicitly deal with the topic of HR analytics in their company and already have experience in its implementation and application.
2. The respective maturity level of the application of HR analytics (reporting, descriptive or predictive) in the company played a subordinate role in order to gather as much experience as possible.
3. The extent of the professional experience with HR analytics of the interviewees also played a minor role, as many firms are only in the early stages of HR analytics.

Each interview was conducted using a semi-standardized guide (see "Appendix"). The basis for this was the previously deductively formed socio-technical framework with the four categories of data, technologies, personnel deployment and organization. The interviews were then transcribed and analyzed using the atlas.ti software. A total of 200 pages of transcribed interview data were collected. The interviews lasted an average of 45 min. The interviews were conducted and transcribed in German. The results of the qualitative content analysis were translated into English.

**Fig. 2:**
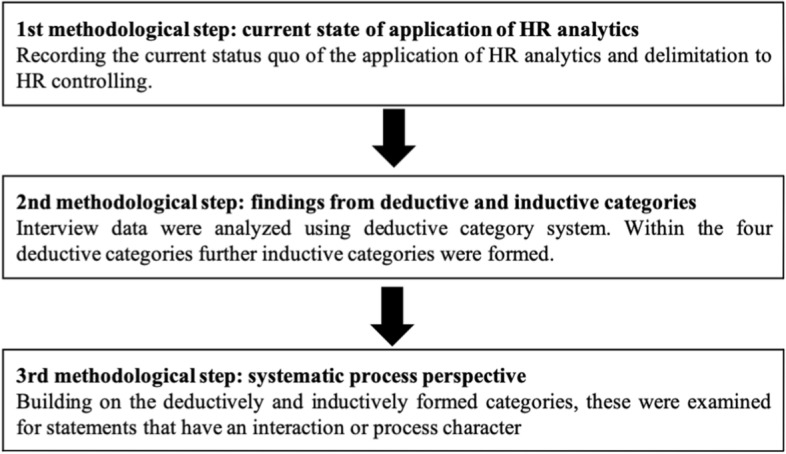
Three-step methodological approach

Figure 2 illustrates our methodological approach. We proceeded in three steps, which are explained in the following. At the beginning of the interview, it was important to capture the interviewees’ understanding of HR analytics, given that there is no uniform definition of HR analytics. Therefore, the interview partners were asked about their task profile and the current state of application of data-driven decisions in their respective firms. This enabled a classification of the status quo in the subsequent analysis of the interviews (see 1st methodological step). Thereupon, specific questions were asked about the current application of HR analytics. In qualitative research, two general approaches can be distinguished: on the one hand, the frequently used inductive approach, which creates a generalization on the basis of specific observations. On the other hand, there is the deductive approach, which tries to transfer generalizations to a specific case (Hyde 2000). We initially have chosen a deductive approach according to Mayring (2014) and a category system was developed on the basis of the factors that have already been derived in our framework as influencing the implementation and application of HR analytics. In a first step, definitions for the individual categories were assigned and suitable anchor examples and coding rules were determined (see Table 2).

**Table 2:** Deductive coding system

Category	Definition	Anchor example	Coding rule
Data	Aspects describing the use of the existing database. Statements about the use of different types of data, as well as problems and challenges, are provided	“On the one hand, the data quality quite simply, that in the HR systems often especially when they so let's say with the payroll data with the attendance-absence data, there is a high data quality. But the further we get into these soft topics, such as skills, where training, qualifications, you have to urge the HR departments that they also maintain them cleanly”	The respective text passage refers to an aspect that describes the use of data. A distinction must be made here as to whether integration into technological systems is being referred to
Technology	Technological systems describe the use of software solutions or other applications of HR analytics. This can be the software itself, but also the interaction with it	“But I just think that it is so still so bogged down how many systems because actually. I talked to two HR business partners once, how many systems they actually have to deal with and they said there are so many different systems. With some, they simply don't know the password anymore and are totally overwhelmed with it. In the end, they just want to have one system, one login and everything in one place”	The respective text passage refers to an aspect that describes the use of software solutions for the application of HR analytics. This can refer to the software solution itself, but also to the use of these solutions by various stakeholders
HR business partner	Aspects that describe the HR business partner in his role within the application and implementation of HR analytics	“Well, we don't want that at all. Yes. So, we don't want to. First of all, we also have the problem that many HR business partners or those who are running around don't necessarily have the analytical capacity and they don't want to deal with it”	The respective text passage refers to the HR business partner within its role in the application and implementation of HR analytics. This can also refer to the "non-application". Statements about the role of the HR function within HR Analytics are also coded
Organization	Intra- and inter-organizational challenges that affect the application and implementation of HR Analytics	“As a rule, however, the biggest hurdles are always first the management attention. That is, that someone from management really wants to drive the whole thing”	The respective text passage refers to an inter- intra-organizational factor that influences the implementation of HR analytics. It is necessary to check whether the statement is technical or does it refer to the own HR function or another classification

The individual interviews were then analyzed and individual text passages could be assigned to the respective categories on the basis of the predefined rules. Based on this, we structured our analysis along the four categories to identify the problems and challenges mentioned by the interviewees. This process allowed us to identify specific aspects within the theoretically developed framework of existing requirements for the implementation and application. In addition to the four deductive main categories of data, technology, HR business partner and organization, further subcategories were inductively formed in the next step. In contrast to deductive coding, inductive coding is based on the principle of open coding. This means that the respective statements of the interviewees are openly coded in the first step and that more aggregated categories emerge from the raw data through repeated examination and comparisons. Ultimately, this allowed for the formation of further subcategories: database, application area, tools for analysis, tools for the provision of the results, current role model, future role model, management support and added value (see 2nd methodological step). Building on the category system, we then specifically searched for patterns of interaction and process within these categories in order to focus on the systematic process perspective (see 3rd methodological step). We proceeded as follows: The interview material was examined one more time for statements describing interaction processes between individual actors in the context of HR analytics. In coding, we defined interaction as the mutual influence between actors. The focus was on the communication and action processes described by the respondents. For example, the following statement describes the provision of analysis results from the HR analytics function to HR business partners.

So the skillset definitely has to grow and we are also in the process of taking the first step and want to make that available as a service, where now the HR business partner, for example, only has read access (Interview 3).

Upon further coding, additional statements could be found that confirmed this process of providing analysis results. In this way, consistent patterns of interaction between different stakeholders could be derived from the individual statements of the interviewees and presented aggregated in the form of a model. This model we derived represents the status quo of HR analytics from a process-oriented perspective.

## Findings

### Current state of application of HR analytics

We begin the presentation of our results with a brief description of the current state of application of HR analytics in the respective firms. In doing so, we highlight the general understanding of HR analytics and address the changes within the processes of HR. In the next step, we present the aggregated findings from the respective categories, which are based on our framework data, technology, HR business partner and organization. Table 3 provides an overview of the 17 interviewees.

**Table 3:** Description of interviewees

Interviewee	Job title	Branch	Maturity level	Used software
1	HR analytics expert	Insurance	Descriptive Predictive	PowerBI SAP analysis plugin
2	HR analytics expert	Industry	Descriptive	Qlik
3	HR analytics expert	Technology	Descriptive	PowerBI
4	HR analytics expert	Mobility	Descriptive Predictive	Tableau, python, R
5	HR analytics expert	Industry	Descriptive Predictive	Python, R, tableau
6	HR analytics expert/Consultant	Industry	Descriptive Predictive	Tableau, python, R
7	HR analytics expert	Industry	Descriptive Predictive	PowerBI, python, R
8	HR analytics expert	Communication	Descriptive	Tableau, celonis
9	Lead HR analytics	E-Commerce	Descriptive Predictive Prescriptive	Tableau, python, R
10	HR analytics expert	Industry	Descriptive Predictive	Tableau, powerBI, R
11	HR analytics expert	Banking	Descriptive Predictive	Tableau
12	HR analytics expert	Technology	Descriptive Predictive	Tableau, python
13	HR analytics expert	Industry	Descriptive	Tableau
14	Consultant HR analytics	Consultancy	–	-
15	Consultant HR analytics	Consultancy	–	-
16	CHRO	Industry	Descriptive Predictive	External HR-analytics software
17	CHRO	Clothing	Descriptive	SAP success factors people analytics

Our analysis of the current state of application resulted in the majority of respondents still being in the early stages of using HR analytics. Many of the firms are currently in a start-up phase, which is characterized by conducting more in-depth descriptive analyses and answering isolated predictive questions. The analysis of how the interviewees define the term HR analytics is characterized by a uniform understanding. HR analytics is a strategic tool that offers the possibility to steer and influence actions and decisions in the HR context on the basis of analyses. The interviewees emphasized that one does not rely exclusively on these analysis results, but that they should be considered as decision-supporting. Rather, the analyses with the help of HR analytics are intended to stimulate the HR business partners to take a deeper look at topics, as one of the interviewees emphasized:

So, there's also a lot of show and tell in the collaboration with HR staff, rather than them sitting down, doing some calculations themselves and thinking afterwards, okay, that'll get us XY. Yes, it's a people business, and it's also a very emotion- and perception-driven business. And then you can use good and relevant analytics to consistently influence people's perceptions and actions (Interview 9).

### Delimitation HR controlling

The goal of HR analytics is to use the methods applied to HR data to check the effectiveness of HR measures and ultimately identify levers that can contribute to improving processes within HR and also the entire company. In particular, the interviewees underlined a demarcation from classic HR controlling. While HR controlling mainly summarizes key figures on historical data and provides relevant groups with information, analyses with the help of HR analytics aim at looking at these key figures in more detail, deepen them and apply them in future-oriented decision-making processes. Our findings underlined that this process is not solely based on predictive HR analytics. In contrast, the interviewees highlighted the potential of descriptive HR analyses, while at the same time emphasizing that it is difficult to manage the next step towards predictive analyses. Additionally, it is found that it can be difficult to draw a line between HR analytics and HR controlling given that the boundaries are sometimes blurred. For illustration, one of our interview partners emphasized that in some cases HR analytics is understood as the automation of reporting processes.

I think it's a big problem. Because then you are actually leading the absurdity of what this three-pillar model is supposed to achieve. And everything that people analytics is supposed to stand for, namely to really bring a benefit to the business and not just to run some purely administrative evaluations somewhere. Yes, definitely. Unfortunately, that's what many organizations have done (Interview 14).

Based on the initial maturity level in which the interviewees find themselves with regard to the implementation of HR analytics in their firms, it can generally be stated that HR analytics is divided into standardized advanced reporting, which has a descriptive character, and project-related questions, which go deeper in the type of analysis. Table 4 presents the results of the qualitative content analysis in condensed form. These results are based on the coding of the interviews and are explained in more detail below.

**Table 4:** Overview of the findings

System	Deductive category	Inductive category	Findings
Technological	Data	Database	Solid data basis often available through HR controlling. Creation of a comprehensive and aggregated HR system as an important requirement
		Application area	Analyses are possible in all functional areas. Recruiting turns out to be particularly predestined. Orientation of analyses in the individual business management context is crucial. On the other hand, especially at the beginning, the process of quantifying all possible processes can help to create a feeling for dealing with data
Technological	Technology	Tools for analysis	The analysis tools are rarely from external providers, as individuality and transparency are criticized here. The necessary competencies are available among the specialists, so that the tool used is not a direct barrier
		Tools for the provision of the results	In addition to the analysis tools, some tools make the analysis results available to other stakeholders. These require a high degree of user-friendliness
Social	HR business partner	Current role model	Insufficient competencies and lack of will and time to acquire them. HR business partners must first create a mindset to work with data in any form. In the long term, respondents do not see the HR Business Partner performing the analyses with HR Analytics. Need for central implementation of HR analytics
		Future role model	Change in the role of the HR business partner. Facilitator and advisor between the HR department, the business departments and HR analytics. Work and decision based on analysis results of HR analytics
Social	Organization	Management support	Management support as a significant factor for the legitimization of HR analytics. In the future, management must demand more work with data and actively integrate it into processes and not just want to introduce the topic for trend reasons
		Added value	The Added-value of analyses using HR analytics represents an important legitimizing factor, but is not captured due to the complexity and novelty of HR analytics

### Technological system: data

#### Database

Our data showed that the data basis is an important challenge for the use of HR analytics. However, it should be noted that firms with a well-functioning HR controlling system have a solid database. These are standard data from the HR management, such as fluctuation figures, sick leave, master data, salaries, etc. One interviewee pointed out that especially firms with little or weak digitization of processes have to struggle with data quality problems. It was pointed out that one of the most important first steps in the implementation of HR analytics should be the creation of an all-encompassing and aggregated HR system, otherwise one is busy with manual data cleansing, especially in the beginning.

If you have an outdated HR system or an old SAP HRM system, you are extremely immobile in the use of the data because you can only get it out with difficulty or not necessarily in the format you would like to have and then you are already back in this operations trap. That is, you come up with a great dashboard, great use case and build it within a week and then you have to download a report every week for the rest of your life, pull it in, maybe clean it up a bit. And these are exactly the pitfalls that you run into (Interview 9).

Our findings showed that firms have a hard time with data integration in particular because HR does not store its data centrally in an enterprise data warehouse, as many firms do out of caution. Consequently, business intelligence topics have also not found access in HR for a long time, which results in a poor-quality database.

#### Application areas

Our analysis reveals that even though HR analytics is applied in different HR functions, all interviewees emphasized that recruiting can be identified as the most effective area. On the one hand, this is due to the large number of data records generated by applications, which makes it possible to develop valid prediction models compared to other application areas. On the other hand, recruiting is also seen as having the greatest potential for highlighting the added value of HR analytics given that it is a major cost factor for many firms. However, it has also been pointed out that rather “new” topics are suitable for data-based analyses, as the attitude toward these newer topics has not yet solidified in the minds of those involved. One interviewee highlighted that this is specifically the case for diversity, i.e. issues such as equal pay, women's quota, and severely disabled quota, as these are high visibility issues where companies are generally more open to learning more.

Because these are fields that have only been established in this way for a few years. And that's where the knowledge has to be built up. So, there is just less gut feeling or felt gut feeling and therefore more room for such analyses (Interview 1).

When using HR analytics, the potential benefits of the individual questions in the application areas should be considered. For instance, one of the interviewees emphasized that the economic benefit should be kept in mind during the analysis.

I [think we] consider far too little in HR analytics or people analytics, that we align ourselves with the business problems (Interview 6).

For a small company with a large turnover, an analysis of the reasons for turnover can create a relatively large added value, whereas on the other hand a large production group, with an identical turnover has a saving that is not significant, but a potential analysis to improve the ergonomic working conditions on the assembly line can increase productivity.

Whereas the orientation of analyses in the business context is crucial, another interviewee also underlined that especially in the beginning the process of quantifying all possible processes in HR management can contribute to developing a feeling for dealing with numbers and can create an orientation to include analyses in the decision-making process.

So everything that is really purely statistical figures first of all in the personnel area. That's good. To be honest, I think it's also important because it helps you to awaken a bit of an affinity for numbers or a feeling for them. But anything that goes beyond a standard evaluation, I would always tie to a concrete business case (Interview 14).

One interviewee also noted that "*you don't want everything you can*" (Interview 12). He emphasized that one must consciously look at whether the analyses make sense for the respective industry and really lead to an increase in effectiveness.

### Technological system: technology

#### Tools for analysis

Besides the data, another technological aspect is the technology itself, i.e. the analysis software used. Here, a largely homogeneous picture emerged among the interviewees. Tools such as Tableau or PowerBI were used for the actual analysis. In rare cases, additional work was done with R or Python. This is mainly the case in firms, which already carry out more advanced analyses. Two firms worked with external analysis tools. One of these is an external software manufacturer that offers a stand-alone HR analytics tool and the other is an integrated analysis function of the HRIS.

Other interviewees were rather critical of the use of external tools, as there is no direct insight into the analysis methods. As an example, tools were cited that offer e.g. speech analyses of interviews that are supposed to predict suitability without providing valid evidence for this. The use of such tools has a counterproductive effect and stirs up fears. Another aspect that has been criticized about external tools is their limited flexibility. An individual adaptation to the circumstances of a company is only possible to a limited extent. Predefined standard use cases may be applicable, but they often cannot be specifically adapted to the particularities of the company structure. This is particularly important in HR management, as it is characterized by a high degree of variance, as the following interviewee noted:

There is a lot of variance in what an organizational structure looks like. Do you have double tops or just single tops, do you have a pyramid or a cell structure? These are all issues that have an extreme influence on the data model that you have to import into such a system. And for the fact that I pay relatively a lot of money for relatively simple dashboards that come out of it, I think that's pretty meager (Interview 9).

#### Tools for the provision of the results

With regard to the question of the technology to be used, a differentiation must be made between the process of the actual analysis and the provision of the results of this analysis. This is strongly related to the understanding of roles in the HR analytics process. This aspect will be discussed in more detail in the course of the results. The presentation of analysis results is provided to the respective addressees via a tool such as Tableau. Here, the interviewees saw the focus above all on ease of use. The simplicity and instinctive approach were emphasized. One of the interviewees pointed out that it is precisely this simplicity that also empowers and motivates people to work with metrics. Furthermore, the flexibility for visual representations was emphasized, which is particularly important for HR management in order to provide HR business partners with a more comprehensible approach to the subject matter.

When choosing the respective tools, it is necessary to define in advance exactly who plays which role in the HR analytics process, as the following interviewee pointed out:

So, we say, you can introduce the best tool if just this, the use of the tool is not clear. So, if the end-user is not clear where he is going to use this (Interview 3).

### Social system: HR business partner

Another aspect in the investigation of our data was the HR business partner. Here we were able to identify the current role of the HR business partner and the problems associated with it. We were also able to identify the extent to which the understanding of the role must change in the future for the effective application of HR analytics.

#### Current role model

If we first look at the statements regarding the competencies of the HR business partners, it became clear that the analytical competencies of the HR business partners were predominantly assessed as poor to barely present. In the eyes of the interviewees, the HR business partner is not the one who carries out in-depth analyses. Our analysis showed that there are three main reasons for this.

First, one of the reasons lies in the nature of HR. The background of working in HR is often different from working with data and key figures, so many of the current HR business partners have avoided basic statistical subjects already in their studies and thus have not developed a connection to data-based analyses in the course of their professional life. Secondly, there is a lack of understanding and rejection of working with data in HR. The third aspect lies in the number of operational tasks and lack of time highlighted by the interviewees. Even if HR business partners are willing to build competencies in the area, this often does not happen due to time constraints.

So it's both the skills they have today and the lack of time to build the skills because they have to deal with „Old Work“ every day (Interview 6).

Even in the long term, the interviewees do not see the HR business partner carrying out the analyses with HR analytics. One interviewee cynically noted that a "*new species of HR employees must first be born*" (Interview 7). Even with advanced competencies in the necessary statistical methods, decentralized performance of analyses by different HR business partners is seen critically. By using different data sets and different methods to conduct the analysis, different people can come to different results: "*there were three different people who gave three different results*" (Interview 8). The interviewees therefore strongly emphasized the need for a central implementation of HR analytics. This can be described as a vicious circle: administrative tasks continue to dominate the task profile of HR employees and thus there is no time for further training in strategically oriented methods such as HR analytics, which should actually provide relief for operational work. Even in the long term, the interviewees do not see that HR employees will be able to conduct analyses on their own.

#### Future role model

Our analysis revealed that the HR business partner will have to take on a different role in the future than that of the analyst. The interviewees emphasized that the HR business partner must develop a stronger sense of working with analytics results in the future. Above all, the interviewees highlighted the function as a mediator and consultant between the HR department, the specialist departments and the HR analytics function. In particular, our findings showed that the implementation of HR analytics is still seen as a centralized independent function. Thereby, the task profile will change due to the closer cooperation with the HR analytics function in the sense that there will be a closer exchange between HR management and the specialist departments. In the future, HR business partners will increasingly take on an advisory role based on the analyses carried out by HR analytics. On the one hand, they should record the requirements of the specialist departments with the help of the necessary HR expertise and communicate these to the HR analytics function in a comprehensible way.

They take the requirements from the business department and then translate them into IT. And in my eyes, something like this is also missing in HR, where a business analyst takes the requirements from HR, so to speak, and then makes them available to the data scientist. That role between the different stakeholders that are involved in the process of a data-based analysis (Interview 15).

We have said that we do not want this analysis as a service, but we would like to participate in the process because we also want to build up the know-how. Yes, and we work together within this framework. We personally have an interpreter function. That is, there are the data scientists who bring the methodology, who build the tool. On the other hand, there is the specialist department, which would like to know what kind of statements are contained in these free-text comments, and we as People Analytics are the interpreters between both worlds and of course also use this for us to build up the know-how (Interview 3).

Our interviewees highlighted, that the future role model must also change in such a way that the old understanding of HR work changes. Working with data and people must not be mutually exclusive in the minds of HR business partners, but must be thought of as a unit. At the present time, this is not yet possible:

Even if it's not explicitly stated: this caveat, when we, when we talk about people, we shouldn't do it in a quantitative way. In terms of feeling, that's something that resonates very often (Interview 1).

### Social system: organization

#### Management support

The interviewees outlined that the role of management is a key aspect of the implementation of HR analytics. When introducing HR analytics on the part of HR, it must be ensured that management is also committed to it.

But it is usually not enough if somehow only one, yes, a sub-department head somewhere says I would like to do the whole thing. Then it fails with a sometime at the latest at the point when the whole thing is presented to the board or something else because they do not consider the whole thing so important yet (Interview 14).

Two key aspects could be identified. First, management plays an important role in legitimizing HR analytics. In-depth analyses are often initiated by management and the results are also fed back to management. However, this also clearly limits the implementation of further analysis projects.

But that is the reason why we have the backing, so to speak, for the projects that we then do. You can look at it the other way round and say that we only do the ones where we have the backing. There aren't many of them, it has to be said (Interview 1).

Secondly, one interviewee also pointed out that management's conviction must also be viewed critically. Trend topics such as data-based analyses in particular only deliver added value if they are understood in their entirety and are not just introduced because it is the latest trend.

But currently, it's really still a lot: this is a trend, I have to jump on it. Data Science in general and the whole artificial intelligence topic is so hyped and there are a lot of articles about it, which the management has picked up somewhere and then they have to do something about it, but just not this, this good understanding of what that actually means for such an HR department or where a department actually stands right now (Interview 8).

In addition to management support, the use of HR analytics also requires management to actively demand work with key figures. This means that management must demand more work with data from HR. This must be done alongside strengthening the understanding of working with data. Demanding analytics results from HR business partners thus additionally contributes to making working with HR analytics more natural for HR business partners. At this stage, the additional involvement is seen as another time factor. It should be noted, however, that according to our analysis the roles in the process of conducting HR analytics are not clearly defined, i.e. the management also does not have a clear contact person for the final analysis results.

#### Added value

One aspect that the interviewees considered particularly difficult to realize in the implementation of HR analytics is the recording of the potential added value respectively the representation of this in monetary key figures. This is particularly difficult because the causal effects of the analyses can only be clearly proven in the rarest of cases. The added value is usually justified, if at all, by time-saving or an increase in employee satisfaction. Another aspect why the added value is not yet captured is the novelty of the topic in the organizations. The interviewees were aware of the need to demonstrate added value, especially to management, but at this stage, they are focusing on conducting analyses. Projects are carried out, which are also approved by the management, since the skepticism is large here, as evidenced by the following statement:

So, the skepticism in this regard is huge. That has to be said very, very clearly. But that is the reason why we have the backing, so to speak, for the projects that we then do (Interview 1).

One interviewee critically noted that the justification and legitimization for the use of HR analytics is flimsy. Data-driven decision-making in HR management is what other corporate functions have had firmly anchored in their structures for many years, and it brings significant benefits there.

Above all, people analytics is a tool to show where inefficiencies are, the alternative to running people analytics is not running it and not knowing what's going on. That's just it, you wouldn't do that in any other area. And in other areas you wouldn't say, do we really need marketing analytics? Of course, you do. And consequently, this retroactive and block position: 'Well, does it really do anything?', I always find a bit flimsy (Interview 9).

To date, none of the interviewees has specifically established a structural process in the sense of tracking the effectiveness of the analysis results and the derived measures.

## Systematic process perspective

**Fig. 3:**
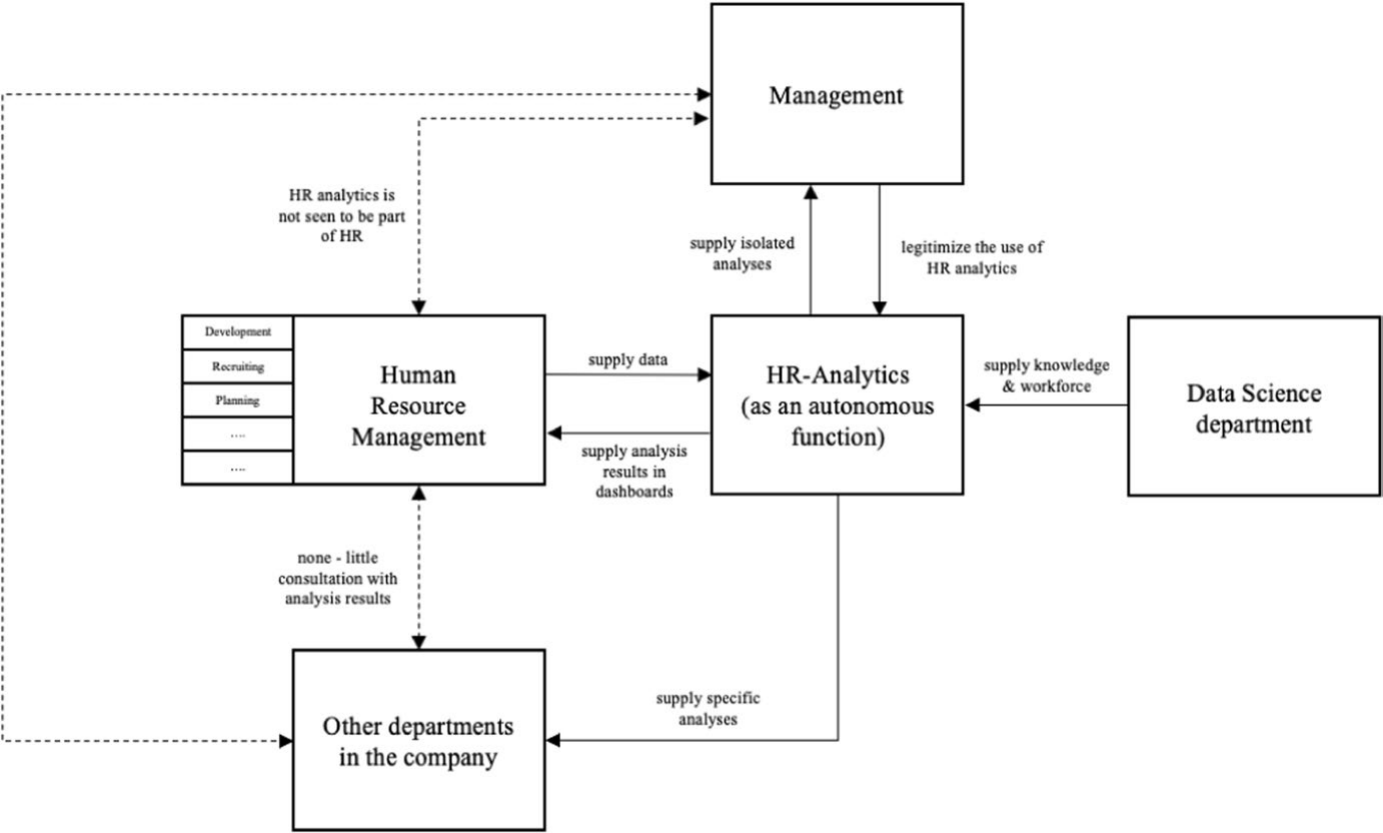
Process model of implementation of HR analytics: status-quo

After examining the results of the qualitative analysis, we also carried out an analysis of the findings from a systematic process perspective. Figure 3 shows the process flow of the implementation and application of HR analytics. Our findings reveal that HR analytics in its more structural anchoring and organizational function is not part of the HR department, but operates autonomously alongside the established HR management as a service provider for various internal customers, including the HR department. When considering the implementation of HR analytics, the first thing that stands out is that most firms tend not to involve HR managers directly in the application. Employees who are responsible for HR analytics are mainly not members of the HR department but belong to departments with a statistical background, such as data scientists, business psychologists or sociologists. Depending on the size of the HR analytics team and the given resources of the respective company, manpower and/or the knowledge from departments with data affinity, e.g. Data Science, are used.

This service character of HR analytics is emphasized by many interviewees and justified, among other things, by the lack of competencies and understanding of numbers on the part of HR managers. The finding of the role of a service provider highlights the critical issue that, in particular in the collaboration with HR, the tasks of HR analytics are not clearly specified. The implications of this missing clarification of responsibilities are twofold. First, it will hinder the successful implementation of tools supporting HR analytics. Secondly, this leads to the added value of the analysis so far not being captured. It is important to define who is ultimately the recipient of the analysis results and who communicates them within the company. The results have shown that the HR analytics function on the one hand directly communicates results to the specific departments or management. The HR management receives these results partly only as information in the form of key figures in a dashboard.

A systematic process for recording the added value of the measures or the implementation of the measures does not exist. A discussion of the results on the part of HR management and the specialist department also often does not take place due to the lack of understanding and access. If we summarize statements regarding existing data, firms with existing HR controlling in particular benefit from historically grown preparatory work. Ultimately, HR analytics at the various levels of maturity often requires the same data as traditional HR controlling. The problems of data management are rather related to the technology factor, as it was pointed out here that especially outdated systems force one to deal more comprehensively with the constant data preparation. The aspect of data in the context of the implementation and application of HR analytics can thus generally be regarded as a factor that takes time but is often given in terms of quantity, insofar as the basic conditions are already present in a company. Angrave et al. (2016) highlight that the HRIS systems in use do not provide the necessary analysis capabilities. This point should be viewed critically, as our results have shown that in the short to medium term, the HR business partner will not be the user of the analysis either. It is also not expected in the long term that the role of the HR business partner will be sharpened in such a way that it can perform and understand data-based analyses. Rather, interviewees see the HR business partner involved in the sense that HR expertise from an HR analytics perspective is needed for targeted analyses. The technology question in this sense does not arise at all from this point of view of the methodological possibilities, but rather how the analysis results can be visually represented by another tool such as Tableau. The actual analysis, carried out by the HR analytics function, will draw on its methodological knowledge and adept programs with its competencies in the analysis.

## Discussion

Falletta and Combs (2021) have noted that despite the interest in the topic of HR analytics, the actual knowledge about it is still in its infancy. This starts with the lack of a common definition and ends with a lack of knowledge about the processes of applying and implementing HR analytics in an intra-organizational context (Falletta and Combs 2021; Greasley and Thomas 2020). The aim of our research was to examine the implementation and application of HR analytics in firms. By applying a socio-technical approach we have developed a theoretical framework which integrates four areas impacting the implementation and application of HR analytics. This framework guided our qualitative research study, which resulted in a more nuanced understanding of the facets of HR analytics as well as its implementation process. The conclusions we have reached from the results of our study will be discussed as follows: first, we will reflect the results of our qualitative study in light of the socio-technical system perspective. From a practical perspective, we then propose a process model for the future application of HR analytics.

### Theoretical implications

In sum, our results showed that there is a common understanding of the future use of HR analytics. This implies the improvement of HR-related decision-making by using a data-driven approach, which aims at achieving strategic business goals. However, a lot of HR analytics analyses are currently in the early stages and are partly project-based or prototype-based. Thereby, firms still face many challenges. This is in line with the findings of Fernandez and Gallardo-Gallardo (2021) emphasizing that firms need to overcome organizational barriers with regard to HR analytics. Our study contributes to this important endeavor by examining the barriers of the social and the technical system:

Our first observation relates to the use of available data and technologies for the application of HR analytics. The availability of data and data integration is considered one of the most important factors in the implementation of HR analytics (Halper 2014; Pape 2016). Our findings confirm this important factor. It should be noted, however, that the interviewees see data integration as a rather operational task, which primarily requires time resources rather than competencies. The provision of the necessary data depends primarily on the level of technologization and the HRIS system used specifically in HR. We did not find any issues regarding the use of external data. However, this may also be due to the fact that many of the respondents are just beginning to explore their options and have not yet conducted analyses that require a deeper data set. It can be noted that the current level of maturity does not meet the demands of some authors who call for a holistic HR analytics function that also includes departments such as finance, production, etc. (Rasmussen and Ulrich 2015; Marler and Boudreau 2017; McIver et al. 2018). The picture is similar for the technology used for analysis: our interviewees have advanced skills, so applying the necessary skills is not a problem. However, it turned out that the issue of technology is much more related to the delivery and visualization of the analytics results rather than to the actual analysis tools. A similar conclusion was also reached by van den Hauvel and Bondarouk (2017). The authors emphasize that HR analytics goes beyond mere analysis and requires convincing visualization and presentation. (van den Hauvel and Bondarouk 2017). Our findings have shown that there is more of a concern with creating awareness that the HR business partner is working with these analytics results and using the dashboards provided (Vargas et al. 2018). It might be argued that the previous data and technology challenges were viewed from the perspective of HR business partners as users of HR analytics. However, our study has shown that they are not the actual people who perform advanced analytics using HR analytics. The HR business partner is much more understood as a customer in this process (Jörden et al. 2021).

We now turn to the discussion of the results of the social system, i.e. the analysis of the role of the HR business partner and intra-organizational context. Previous research has identified two different approaches to embedding HR analytics in the organization. On the one hand, HR analytics is seen as an independent function that uses the potential of data scientists (Rasmussen and Ulrich 2015). On the other hand, it is argued that an integration of HR analytics in HR management is worthwhile (Bassi 2011; Falletta and Combs 2021).

Our results have shown that the preferred path of the firms in our study is the former and that a new stand-alone HR analytics function is emerging alongside the HR function itself. This may be due to the fact that analytical skills in HR are not sufficient to apply HR analytics independently (Angrave et al. 2016; Marler and Boudreau 2017). Although our results have shown that HR itself can also be identified as the initiator of HR analytics, the function does not emerge within HR but is carried out by individuals who inherently bring the necessary competencies. This approach can be promising but runs the risk of leaving out HR managers who have been active to date. Ultimately, this can lead to competencies being built up in silos in the long term and not being taught throughout the HR sector. To prevent this, a holistic approach is needed. HR analytics should not be considered as a stand-alone function of the HR value chain but should map it across the entire functions of the HR value chain.

Another important factor that plays a role is the recording of the added value of HR analytics. Our findings have shown that many analytics results are communicated to the respective addressees, e.g. the specific departments, HR or management without an accurate evaluation of the effectiveness or efficiency of these measures afterward. However, one of the core objectives of HR analytics is to improve organizational productivity and employee experience (Tursunbayeva et al. 2018). This lack of evaluation also means that HR management's own strategic strengths fall short of expectations and thus the reasons for the legitimacy of HR analytics cannot be optimally communicated to management (Bassi et al. 2012). As shown in our status-quo process model (see Fig. 3), HR analytics is currently acting autonomously as a service provider. This finding is in line with the observations of Jörden et al. (2021), who also found in an ethnological study of a people analytics team that HR analytics was “primarily driven and restricted by customer requirements, and as a consequence PA as a specialist professional HR practice was undermined by a lack of managerial commitment to technical quality “(Jörden et al. 2021: 11). Especially management as a customer is a double-edged sword: our study has shown that the legitimization of HR analytics is a decisive factor in the implementation of HR analytics. On the other hand, Jörden et al. (2021) see management as a critical factor in this respect, as it can also undermine the possibilities of HR analytics by only carrying out analyses that are demanded by management.

Moreover, we know little about which measures are ultimately implemented by the individual addressees from the derived analysis results (Ellmer and Reichel 2021). The lack of evaluation of the derived measures leads to the fact that the seemingly relevant business context of the analyzed issue cannot be clearly evidenced (McIver et al. 2018). In the future, HR analytics must have more legitimacy grounds than those of management. This means closer cooperation between firmly integrated functional areas and HR so that HR analytics does not become an end in itself and can bring the promised added value (Rasmussen and Ulrich 2015).

In summary, it can be stated that there is a need to better understand HR analytics from a process perspective. This implies to define the different internal stakeholders which are involved in the HR analytics process and to cover their respective ideas and wishes. In line with Ellmer and Reichel (2021) our findings show that the role of the HR business partner needs to be defined more clearly in the future. Also, the required understanding and affinity for the work with data and data-based analyses can only succeed if the scope of responsibility of the HR department is clearly defined. A sharpening of the role of HR can help to clarify whether HR analytics promises the actual added value, i.e. an increase in HR’s strategic alignment in the organizational context (Greasley and Thomas 2020). Still, it is difficult to implement a purely autonomous execution of HR analytics without the involvement of traditional human resource management (Bassi 2011). Thus, the targeted communication of the necessary competencies and the definition of the areas of responsibility for HR analytics is a necessary step that firms will have to take to ensure the effective application of HR analytics. In this regard, Falletta and Combs (2021) don’t position HR analytics as a separate function but argue that it should be located directly in HR management. This contradicts previous research which concluded that HR analytics should be a permanent function (Rasmussen and Ulrich 2015; Ulrich and Dulebohn 2015).

### Managerial implications

**Fig. 4:**
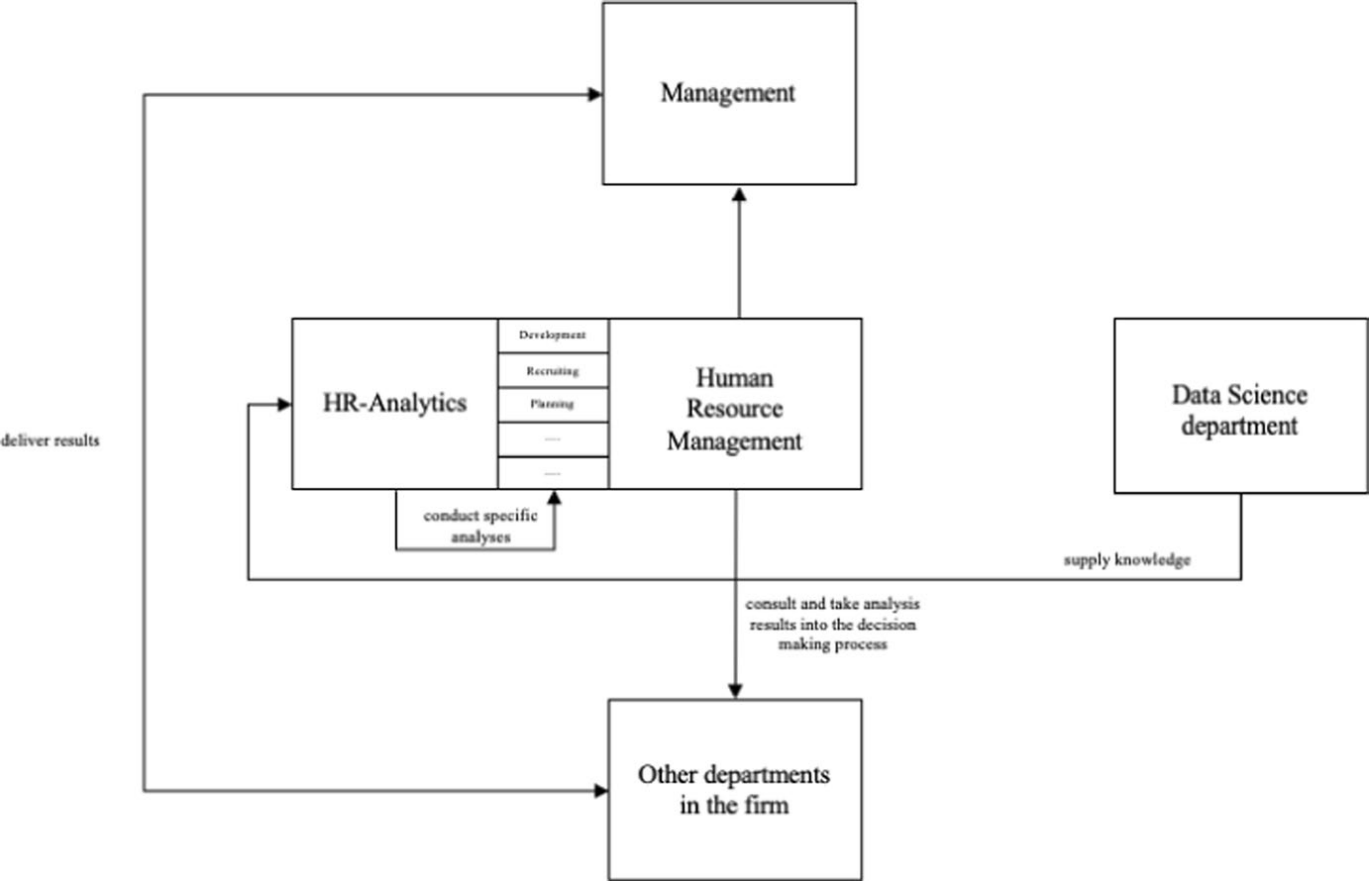
Supposed process model for the future of implementation of HR analytics

In light of our findings, we argue that in order to strengthen the practical implementation and application of HR analytics the following aspects can be defined as adjusting screws from a process perspective: At a social level, there is a need for a clearer clarification of roles in the intra-organizational process of HR analytics. At the technical level, one needs to be aware that adapted software solutions have to fit the respective competencies of the HR business partners. Based on this we propose a process model for the future application of HR analytics (see Fig. 4). Our study has shown that it is not necessary for an HR business partner to have the skills to carry out analyses independently. Rather it is crucial to develop an awareness and speak the language required to understand these analyses and discuss them with the other departments in the next step. A discussion of whether the users of HR analytics have a statistical and business background or rather a social, behavioral and organizational sciences background (Falletta and Combs 2021) is not expedient. The following applies here: many solutions lead to the goal; ultimately, it is important that the analyses are based on valid methods and are goal-oriented from the perspective of HR management. In order for an HR business partner to be able to work with these analysis results, more user-friendly software solutions will be needed in the future. Again, it is not a question of carrying out the analysis itself, but rather of providing the necessary information in a targeted manner in order to enter into an exchange with the specialist departments. The role of the HR business partner as a future consultant is seen as particularly important as the previous way of communicating the results of the analyses is not precisely defined: If it is possible for the HR department to make decisions in cooperation with the specialist department on the basis of analysis results, this is also reflected in the communication with management. This in turn has a positive effect on management's attitude toward HR. In the long run, the closer integration of HR analytics and the HR business partners can lead to achieving the actual goal of strengthening the strategic role of HR. For these reasons, we advocate a multidimensional role model for the application of HR analytics.

## Limitations

The study has some limitations. First, the results are based on the conduct of qualitative research. This methodological approach of qualitative research naturally entails some limitations. The results obtained cannot be generalized to a broader population with the same degree of generalizability. However, statistical analysis of the results is also not intended, as the goal of qualitative research should be to gain new insights based on experience and detailed descriptions. We recommend that our proposed model of HR analytics implementation be tested in the future using descriptive and observational studies. Since HR analytics is an interface function between HR and business informatics, one possible approach would be to apply design science research from business informatics. The approach starts from an application-oriented problem, on the basis of which an IT artifact is created and tested in several iterative steps. Future research could target approaches here and investigate different IT artifacts to explore the implementation and application of HR analytics more deeply empirically.

Second, the findings of our study are based on interviews with HR analytics experts. These experts have been chosen according to a clearly defined set of criteria. Given that the implementation and application of HR analytics is still in its infancy in most of the firms, the knowledge base of interviewees is at a beginner or mediocre level. To gain more insights, we recommend to analyze the ongoing development of HR analytics and its structural positioning within organizations once the application and implementation have become more deeply established in firms. This would enable further exploration of the role of the HR business partner as well as identify strategies of how to close the gap between HR analytics and HR function. In addition, we did not examine any other stakeholders involved in the process (e.g., management, HR business partners or specialist departments) as part of this study. In order to get a more holistic picture of the model proposed by us, we recommend conducting further interviews with these groups as well. As a final note, our study was only conducted in German-speaking countries, so the use of HR analytics must be reflected under the applicable data protection aspects.

Third, as mentioned at the beginning, the COVID 19 pandemic has given a new boost to the topic of HR analytics. The sharp increase in the use of digital technologies and the changes in working conditions offer a wide range of new opportunities for analyzing HR issues. Since this study took place precisely during the pandemic and the impact of the pandemic was not a focus, future research should investigate the extent to which the new circumstances influence the implementation and application of HR analytics. In addition, it should be quantitatively investigated to what extent HR analytics could be refined based on the multitude of new types of data sources resulting from the new types of working conditions.

## Conclusion

Firms have recognized the opportunities presented by HR analytics; however, it is challenging for HR to convert their available data (sources) into meaningful strategical value. Moreover, scant research has explored how the implementation and application of HR analytics is achieved. This study provides one of the first attempts to examine the socio-technical aspects that underline the process of HR analytics. The results of this study contribute to the existing literature by showing that the function of HR analytics needs to be reconsidered. Also, it encourages future research to dive deeper into the variety of contextual and process conditions strengthening or weakening the value of HR analytics.

## Appendix

### Interview guide

#### Application

1. What do you understand by the term HR analytics?
2. Where does it differ from classic HR controlling?
3. What concrete added value does the use of HR analytics offer?

- Can you quantify the added value using key figures? (e.g. resource savings, quality of recruitment, more transparency, etc.).
  If not: How would you describe the added value subjectively?

4. What has changed in the HR department as a result of using HR analytics? What has changed from an overall organizational perspective?
5. Can you identify specific areas where data-driven decisions have increased?

- What was the cause of this exact area being supported by data analytics? What exactly has changed about the process? How did this decision take place in the past? Have there been any advantages or disadvantages to this?

### Data

1. What problems were/are there in general with the evaluation of HR data?

- On the technical/data level
- On the evaluation level

2. What are the data sources for the HR data used?

- How is this data systematically collected?
- Who has access to this data?
- Are there any problems regarding interfaces in data collection?

3. Which specific questions are answered with the help of the data analysis? Why these in particular?

- Which data are necessary for this?

4. How do you collect and analyze specifically qualitative data?
5. Do you include external data in the analyses, if so which ones? What difficulties do you encounter in doing so?

### Technology

1. What software do you currently use to analyze HR data? Dedicated HR analytics software or individual tools in different application areas?
2. What skills does it take to use this software/tool? Did you already have these skills or were they obtained elsewhere?
3. What difficulties are encountered with this software/tool? How could these be improved?

### HR business partner

1. Who has been the primary initiator of HR data analysis to date? Why?
2. What does the power user of HR data analysis look like? Individual person or a data team?
3. What are the advantages and disadvantages of this?
4. What other stakeholders are involved in the process?
5. At which interfaces do difficulties arise? Which ones?
6. Has the use of HR analytics changed the collaboration with other departments/areas? In what form? If not, would it have to change from your point of view?

### Organization

1. What organizational difficulties have arisen in the analysis of HR data? How were/are they dealt with?
2. What difficulties have arisen in the analysis of HR data on the part of the employees? How was/is this dealt with?
